# Responders to Wide-Pulse, High-Frequency Neuromuscular Electrical Stimulation Show Reduced Metabolic Demand: A ^31^P-MRS Study in Humans

**DOI:** 10.1371/journal.pone.0143972

**Published:** 2015-11-30

**Authors:** Jennifer Wegrzyk, Alexandre Fouré, Yann Le Fur, Nicola A. Maffiuletti, Christophe Vilmen, Maxime Guye, Jean-Pierre Mattei, Nicolas Place, David Bendahan, Julien Gondin

**Affiliations:** 1 Aix-Marseille Université, CNRS, CRMBM UMR 7339, Marseille, France; 2 APHM, Hôpital Sainte-Marguerite, Service de Rhumatologie, Pôle Appareil Locomoteur, Marseille, France; 3 Human Performance Lab, Schulthess Clinic, Zurich, Switzerland; 4 ISSUL, Institute of Sport Sciences, Faculty of Biology and Medicine, University of Lausanne, Lausanne, Switzerland; 5 Department of Physiology, Faculty of Biology and Medicine, University of Lausanne, Lausanne, Switzerland; University of Sydney, AUSTRALIA

## Abstract

Conventional (CONV) neuromuscular electrical stimulation (NMES) (i.e., short pulse duration, low frequencies) induces a higher energetic response as compared to voluntary contractions (VOL). In contrast, wide-pulse, high-frequency (WPHF) NMES might elicit–at least in some subjects (i.e., responders)–a different motor unit recruitment compared to CONV that resembles the physiological muscle activation pattern of VOL. We therefore hypothesized that for these responder subjects, the metabolic demand of WPHF would be lower than CONV and comparable to VOL. 18 healthy subjects performed isometric plantar flexions at 10% of their maximal voluntary contraction force for CONV (25 Hz, 0.05 ms), WPHF (100 Hz, 1 ms) and VOL protocols. For each protocol, force time integral (FTI) was quantified and subjects were classified as responders and non-responders to WPHF based on k-means clustering analysis. Furthermore, a fatigue index based on FTI loss at the end of each protocol compared with the beginning of the protocol was calculated. Phosphocreatine depletion (ΔPCr) was assessed using ^31^P magnetic resonance spectroscopy. Responders developed four times higher FTI’s during WPHF (99 ± 37 ×10^3^ N.s) than non-responders (26 ± 12 ×10^3^ N.s). For both responders and non-responders, CONV was metabolically more demanding than VOL when ΔPCr was expressed relative to the FTI. Only for the responder group, the ∆PCr/FTI ratio of WPHF (0.74 ± 0.19 M/N.s) was significantly lower compared to CONV (1.48 ± 0.46 M/N.s) but similar to VOL (0.65 ± 0.21 M/N.s). Moreover, the fatigue index was not different between WPHF (-16%) and CONV (-25%) for the responders. WPHF could therefore be considered as the less demanding NMES modality–at least in this subgroup of subjects–by possibly exhibiting a muscle activation pattern similar to VOL contractions.

## Introduction

Neuromuscular electrical stimulation (NMES) has gained popularity in the fields of sports and rehabilitation and is widely used in patients [1] and healthy subjects [2, 3] with the aim of enhancing, preserving or restoring skeletal muscle mass and function. Conventionally, electrical stimuli with short duration pulses (50–400 μs) are delivered at frequencies ≤ 50 Hz with relatively high current intensities (hereafter referred to as conventional (CONV) NMES) [4]. It has been shown that these stimulation parameters elicit contractions primarily through the direct activation of motor axons, due to both the susceptibility of motor axons to short pulse durations and the antidromic collision at high stimulation intensities [5]. Contrary to voluntary (VOL) contractions, CONV does not recruit motor units according to the size principle (i.e., orderly recruitment with fatigue-resistant motor units being recruited first) [6, 7]. CONV leads to random motor unit activation [7–10] thereby resulting in the recruitment of both slow and fast motor units even at relatively low force levels [7, 10]. Because CONV also results in a spatially-fixed and synchronous activation pattern, it has been shown to induce a higher energetic demand and thus a faster onset of muscle fatigue than VOL exercise [11, 12], the major drawback of this stimulation protocol.

The use of high-frequency NMES (100 Hz) with wide pulses (1 ms) (wide-pulse high-frequency, WPHF) could enhance the reflexive contribution to motor unit recruitment so that motor units would be recruited in a more natural manner according—or in resemblance—to the size principle [4, 5]. It has been demonstrated that sensory axons exhibit a longer strength duration time constant and a lower rheobase (i.e., minimal current amplitude that results in membrane depolarization) as compared to motor axons and are therefore preferentially activated with long stimulation pulses [13, 14]. Additionally, the low current intensities applied during WPHF minimize antidromic collision [5, 15]. Evidence in favor of the assumption that changes within the synaptic pathway contribute to a larger extent during WPHF as compared to CONV was provided by nerve block experiments and electromyographic (EMG) activity measurements showing the presence of H- (i.e., Hoffmann) reflex and asynchronous activity [16–18]. Furthermore, contrary to CONV, low-intensity contractions evoked by WPHF (≤ 10% of maximal voluntary contraction (MVC) force) can produce gradually increasing forces for a given stimulation intensity. This has been attributed to the progressive recruitment of fatigue-resistant motor units via afferent pathways [5, 17, 19] and is commonly referred to as “extra force” (EF) [17], i.e., the force that arises in addition to what would be expected from the direct response to motor axons stimulation. This EF phenomenon is frequently associated with central mechanisms such as posttetanic potentiation at the Ia synapse, temporal summation of subthreshold excitatory postsynaptic potentials of Ia fibers to the motoneurons and activation of persistent inward currents in spinal motoneurons [4, 17, 18, 20]. Recently, the force evoked by WPHF has been found to be highly variable between individuals [21, 22]. Subjects were therefore classified as responders (showing EF) or non-responders (showing no EF) to account for the high inter-individual variability. Interestingly, in this previous study only the responder subjects exhibited a depression of the H-reflex response following WPHF [22], thus suggesting a spinal involvement.

Nevertheless, the central origin hypothesis associated with WPHF has been recently challenged by Frigon et al. [23]. In the latter study, anaesthetic nerve block experiments in human subjects and nerve transection in decerebrate cats failed to abolish the increment in evoked force, while changes in muscle length affected the EF. The authors therefore suggested that EF may be explained by peripheral rather than central factors, i.e., intrinsic muscle properties such as Ca^2+^ release, sensitivity and/or phosphorylation of myosin light chains [23]. Also, increased stimulation frequency was reported to induce a higher metabolic demand [24, 25] thus challenging the hypothesis that WPHF could lead to a lower energy demand than CONV. Moreover, fatigue occurrence defined as “a failure of the neuromuscular system to maintain the required force” [26] has been recently shown to be greater for repeated WPHF trains based on the observation that the decline in evoked force was significantly higher as compared to CONV at least when responders and non-responders were analyzed together [27].

Until now, ^31^Phosphorus-magnetic resonance spectroscopy (^31^P-MRS) studies have shown that, due to the specific temporal and spatial recruitment of muscle fibers, CONV induced greater phosphocreatine (PCr) depletion and higher acidosis as compared to VOL contractions performed at the same relative force level [12, 28, 29]. The present study was designed to compare the metabolic demand associated to CONV, WPHF and VOL contractions using ^31^P-MRS. According to the hypothesis that EF are related to changes within the synaptic pathway, we suggest that for repetitive intermittent contractions at a given initial force output, responders to WPHF would exhibit a lower metabolic demand than CONV thereby approaching the metabolic profile of VOL. This could provide additional evidence in favor, or against, potential differences in motor unit recruitment between CONV and WPHF.

## Materials and Methods

### Subjects

Eighteen healthy subjects (13 men and 5 women, age: 29 ± 7 years, weight: 67 ± 7 kg, height: 173 ± 9 cm, mean ± SD) participated in the study. They occasionally performed physical exercise but none of them participated in regular and competitive training. All subjects were asked to refrain from intense physical activity 48 hours prior to their visits to the laboratory. They gave their written informed consent to take part in the study, which was approved by the Local Human Research Ethics Committee Sud Méditerranée I (n° 2012-A01265-38) and conformed to the Declaration of Helsinki.

### Study design

Participants were asked to visit the laboratory for the familiarization session (first visit) and for the actual ^31^P-MRS session (second visit) with an interval of 3–7 days in between. During the familiarization session, MVC force was first assessed and then the individual current intensity was determined using electrically-evoked testing trains (as described below). Five submaximal (10% MVC) isometric contractions of 20 s were then completed per each protocol (CONV, WPHF, VOL). The ^31^P-MRS testing session lasted ~2 h and comprised 1) a warm-up consisting of 5–7 submaximal plantar flexions of 5 s, 2) the assessment of isometric MVC force, 3) the adjustment of NMES intensity by using 2-s testing trains and 4) the three exercise protocols (i.e., CONV, WPHF, VOL) each comprising 20 contractions of 20 s which were performed in a randomized order across subjects.

### Experimental setup

Two flexible surface electrodes of 5 × 13 cm and 5 × 9 cm (Stimex, Schwa-medico GmbH, Ehringshausen, Germany) were placed on the right *triceps surae*. The proximal (larger) electrode was placed over the gastrocnemii muscles at approximately the point of their largest circumference whereas the distal electrode covered the soleus below the bottom of the gastrocnemii muscle belly [4, 17]. While lying in a supine position on the MR scanner bed, the subjects’ right knee was fixed at ∼170° (almost extended position) and the forefoot and heel were fastened by a belt around the ankle to a home-built MR compatible ergometer. The ergometer consisted of a foot pedal coupled to a force transducer (sensitivity: 4.5 mV/N) with its amplifier (gain: from 0.87 to 0.42 mV/V; Sensorex 3310, Archamps, France) to measure isometric plantar flexion force. The foot was securely held in position with an ankle angle of 90° while the thigh and the hips were firmly fixed to the bed to limit force generation by muscles other than the plantar flexors.

#### Assessment of MVC force

Subjects performed three to five MVCs of approximately 5 s until the highest MVC force could not be further increased. Each contraction was separated by 2 min of rest. Participants were asked to fold their arms over the chests and to concentrate on contracting exclusively the plantar flexor muscles. Force signal was recorded at a sampling frequency of 1 kHz using Powerlab 16/36 data acquisition system and software (LabChart 7, ADInstruments, Sydney, Australia).

#### Electrically-evoked testing trains

At the beginning of each protocol, 2-s testing trains with characteristics similar to respective NMES protocols (see section below), were applied by gradually adjusting stimulation intensity in an attempt to reach a force level corresponding to 10% MVC (tolerance range: 8.5–11.5% MVC). Electrically-evoked contractions generating similar force levels were previously reported to minimize antidromic collision [15, 18].

#### Protocols

Each exercise protocol consisted of 20 repetitions of 20-s isometric plantar flexions at 10% MVC separated by rest periods of 20 s. Monophasic rectangular pulses were delivered using a constant-current stimulator (Digitimer DS7A, Hertfordshire, UK; maximal voltage: 400 V) at 100 Hz (pulse duration: 1 ms) and 25 Hz (pulse duration: 0.05 ms) for the WPHF and CONV protocols, respectively [27]. Subjects were consistently instructed to relax their plantar flexor muscles during NMES. For the VOL condition, subjects were provided with a visual feedback to maintain the voluntary force at an intensity of 10% MVC and were instructed to comply with the timing of the contraction, i.e., an exercise:rest cycle of 20:20 s, as indicated by a pop-up window next to the force output diagram.

#### ^31^P-MRS measurements

^31^P-MR spectra were acquired using a 1.5T Siemens magnet (MAGNETOM Avanto, Siemens AG, Erlangen, Germany). A ^31^P-^1^H surface coil (^1^H coil: diameter of 275 mm, ^31^P coils: 120 x 140 mm loop and 240 x 120 mm butterfly coil, Rapid Biomedical GmbH, Rimpar, Germany) was placed directly on the skin to cover the midportion of the right *triceps surae*. A set of multi-slice fast proton MR images was initially recorded to determine the position of the calf muscle with respect to the surface coil. Then an automatic localized shimming procedure was used to optimize the magnetic field homogeneity. Resting full relaxed spectra were acquired at the beginning of the experimental procedure with a repetition time of 15 s (Number of excitation: 4). ^31^P-MR spectra were then recorded during a 20-s rest period and subsequently during the exercise protocols with the following parameters (radio frequency hard pulse duration: 500 μs, repetition time: 2 s, Number of excitation: 1, sweep width: 32 kHz, data points: 4096, dwell time: 128 ms, flip angle: 90°). The MR acquisition was synchronized to the stimulation procedure using the Powerlab system. For each subject, all ^31^P-MRS protocols were performed on the same day with identical positioning of the stimulation electrodes and the MRS coil. The three protocols were separated by 15 min of recovery and were randomized to minimize any possible fatigue effect.

### Data analysis

#### Mechanical traces and classification of responders and non-responders

MVC force was quantified as the maximal peak force achieved across the different trials. Force time integral (FTI) was quantified for each contraction and then summed together for each protocol. The FTI was also summed every 4 contractions (i.e. C4, C8, C12, C16, C20) to assess the kinetics of force production during each protocol. The mean force was calculated as the average force for all 20 contractions relative to the MVC value for each subject (% MVC). Furthermore, a fatigue index was calculated as follows: fatigue index = [(FTI C20—FTI C4) / FTI C4] × 100.

On the basis of the difference between the FTI of WPHF and CONV for each train, i.e., DELTA FTI = FTI_WPHF_-FTI_CONV_, we applied a k-means cluster approach that has been recently performed to differentiate responders from non-responders with respect to EF [22]. By calculating the difference in FTI between the NMES protocols we aimed at differentiating moderate force increases in response to CONV (most likely linked to intrinsic muscle properties) from the actual WPHF-evoked EF.

#### ^31^P-MRS

**Fig 1:**
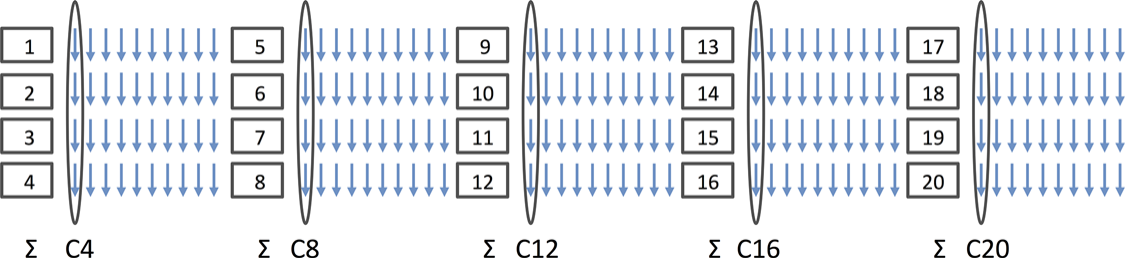
Summation of the first spectra for four subsequent trains in order to improve spectral resolution and to assess PCr depletion over time (i.e. five time-points, i.e., contraction C4; C8; C12; C16; C20). Each rectangle represents one contraction of 20 seconds, each arrow one MR spectrum acquired from 2 sec after each contraction.

Due to stimulation artifacts during the NMES-induced contractions, the spectra for each protocol were only analyzed during the resting intervals of the 20 contractions. In order to obtain an adequate spectral resolution for the phosphorylated compounds and to avoid metabolic changes due to recovery, only the first spectrum immediately after each contraction was considered. All these spectra were then averaged for four contractions each (Fig 1). This analysis allowed to monitor the kinetics of the metabolic changes during each protocol.

Relative concentrations of PCr, inorganic phosphate (Pi) and adenosine triphosphate (ATP) were obtained by a time–domain fitting routine using the AMARES algorithm [30] interfaced by CSIAPO [31] with an initial time resolution of 2 s and appropriate prior knowledge of the ATP multiplets. Considering a 8.2 mM ATP concentration [32], the resting absolute concentrations of both PCr and Pi were expressed as the average of the 7 spectra prior to each protocol in relation to the fully-relaxed value obtained at the beginning of the experimental procedure. Intracellular pH was calculated from the chemical shift difference between the Pi and the PCr signals [33]. The relative PCr depletion (% of resting values) was evaluated for five time points according to the average value of four contractions each (i.e., C4, C8, C12, C16, C20) in order to assess the PCr-kinetics for each protocol. Absolute PCr depletion (i.e., ∆PCr in mM) was also normalized to the corresponding FTI at each time point. The related ∆PCr/FTI ratio was considered as an index of the metabolic demand [34] and based on the linear model from Meyer [35] for submaximal work stating that intramuscular PCr changes are proportional to oxygen consumption and ATP hydrolysis. The difference between resting pH and end-protocol pH values (∆pH, average value of all 20 spectra) was calculated.

#### Statistical analysis

Statistical analyses were performed using Statistica software (Stat-Soft 9, Tulsa, OK, USA). Statistical power was tested *a posteriori* for metabolic demand (ΔPCr/FTI ratio) of 1) WPHF for the responders vs. non-responders and 2) for WPHF vs. CONV in the responders only. Clustering on EF was performed for all 20 contractions per individual. Normality was checked before each analysis using Kolmogorov-Smirnov test. Unpaired t-tests were used to assess differences in MVC force and mean current intensity required to obtain 10% MVC between responders and non-responders. A two-way ANOVA [group (responders, non-responders) × protocol (CONV, WPHF, VOL)] with repeated measures on the second factor was performed on total FTI, mean force, resting PCr, resting Pi, resting Pi/PCr, delta PCr, end-protocol Pi/PCr, fatigue index and ΔpH. Three-way ANOVA [group (responders, non-responders) × protocol (CONV, WPHF, VOL) × contraction (i.e., C4, C8, C12, C16, C20)] with repeated measures on the last two factors was performed on FTI values, PCr concentrations (including C0, i.e., resting values) and ΔPCr/FTI ratio. When a main effect or a significant interaction was found, Newman’s Keuls post hoc analyses were performed. Data are presented as mean ± SD in text and tables and as mean ± SE in figures. Significance was accepted for P < 0.05.

## Results

### Force output and classification of responders vs. non-responders

**Fig 2:**
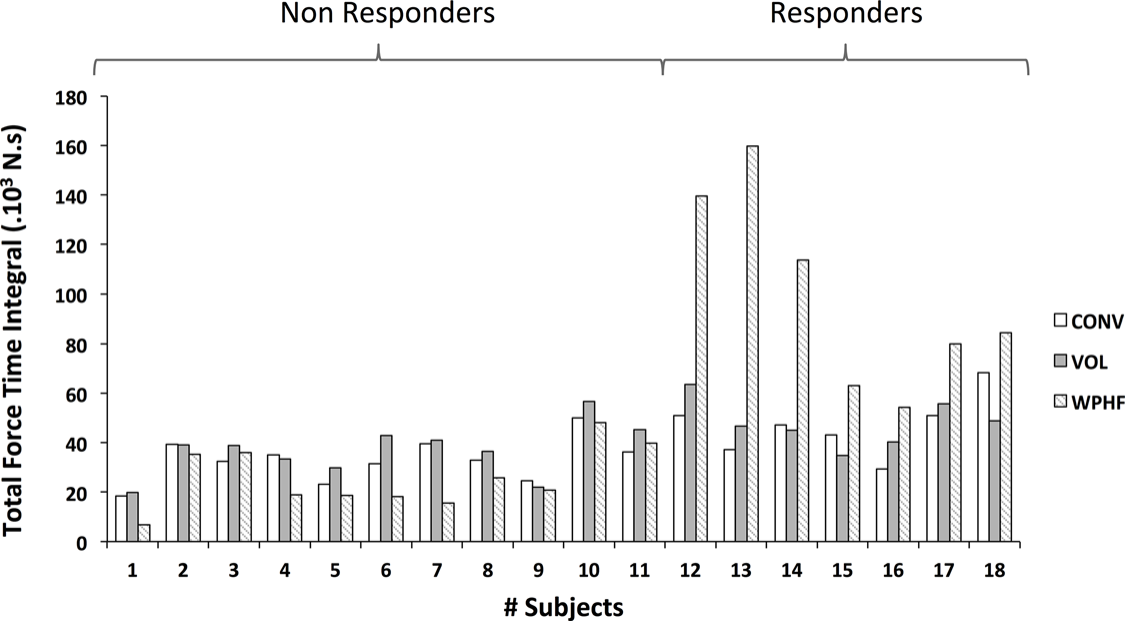
Individual force profiles for all 18 subjects and for each protocol calculated as the sum of the force time integral (FTI) for all 20 contractions. K-means analysis of Extra Forces resulted in the classification of 11 non-responders and 7 responders for the latter of which the FTI was significantly higher for WPHF NMES as compared to the other two exercise modalities. Note the high inter-individual variability of FTI for WPHF NMES.

As illustrated in Fig 2, the total FTI for WPHF showed a high variability between individuals. According to the k-means clustering analysis 7 subjects (6 men /1 woman) were identified as responders to WPHF whereas 11 subjects (7 men / 4 women) were classified as non-responders. MVC values were significantly higher (P < 0.05) for responders (1232 ± 206 N) than for non-responders (949 ± 217 N); this could be explained by the fact that the proportion of men was higher within the responders.

For the responder group, the total FTI was significantly higher (P < 0.05) for WPHF (99 ± 37 ×10^3^ N.s) when compared to both CONV (47 ± 11 ×10^3^ N.s) and VOL (48 ± 9 ×10^3^ N.s). The non-responder group showed no significant difference in total FTI between the three conditions (WPHF: 25.8 ± 11.8 ×10^3^ N.s; CONV: 33.0 ± 8.4 ×10^3^ N.s; VOL: 36.8 ± 9.9 ×10^3^ N.s). The total FTI for WPHF was significantly higher in the responders as compared to the non-responders. Accordingly, in the responder group the mean force for WPHF (19.9 ± 3.7% MVC) was higher compared to both CONV (9.6 ± 1.9% MVC) and VOL (9.7 ± 0.4% MVC) and was also higher compared to non-responders (WPHF: 6.7 ± 3.0%). No difference in mean force between protocols was found in non-responders (CONV: 8.8 ± 1.3% MVC; VOL: 9.7 ± 0.4% MVC).

**Fig 3:**
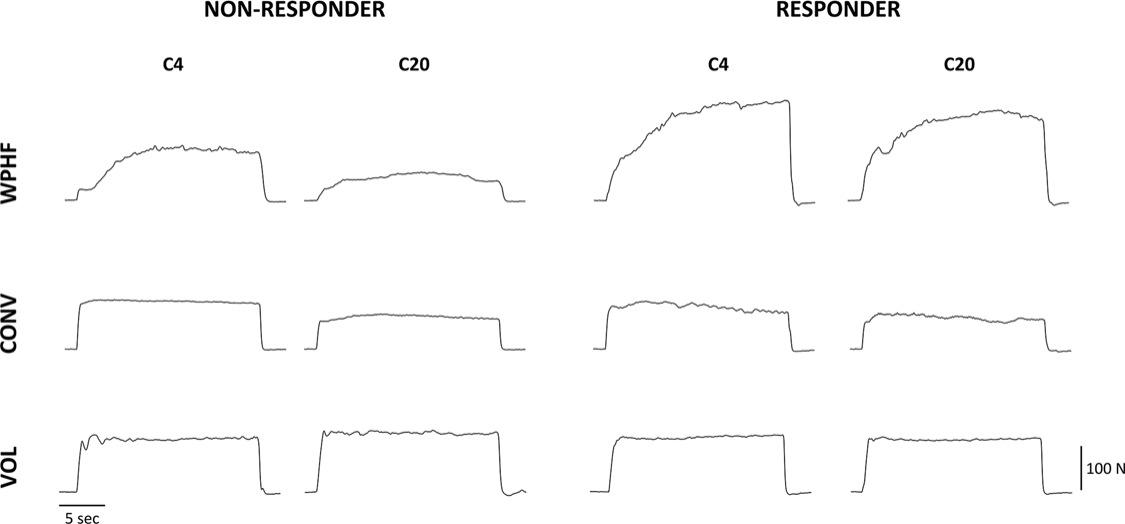
Representative force generation profiles during the first four (C4) and the last four (C20) contractions of the WPHF, CONV and VOL protocols for one non-responder (A) and one responder subject with similar MVC values Note that force production was higher and fatigue was lower for the responder as compared to non-responder during the WPHF protocol.

**Fig 4:**
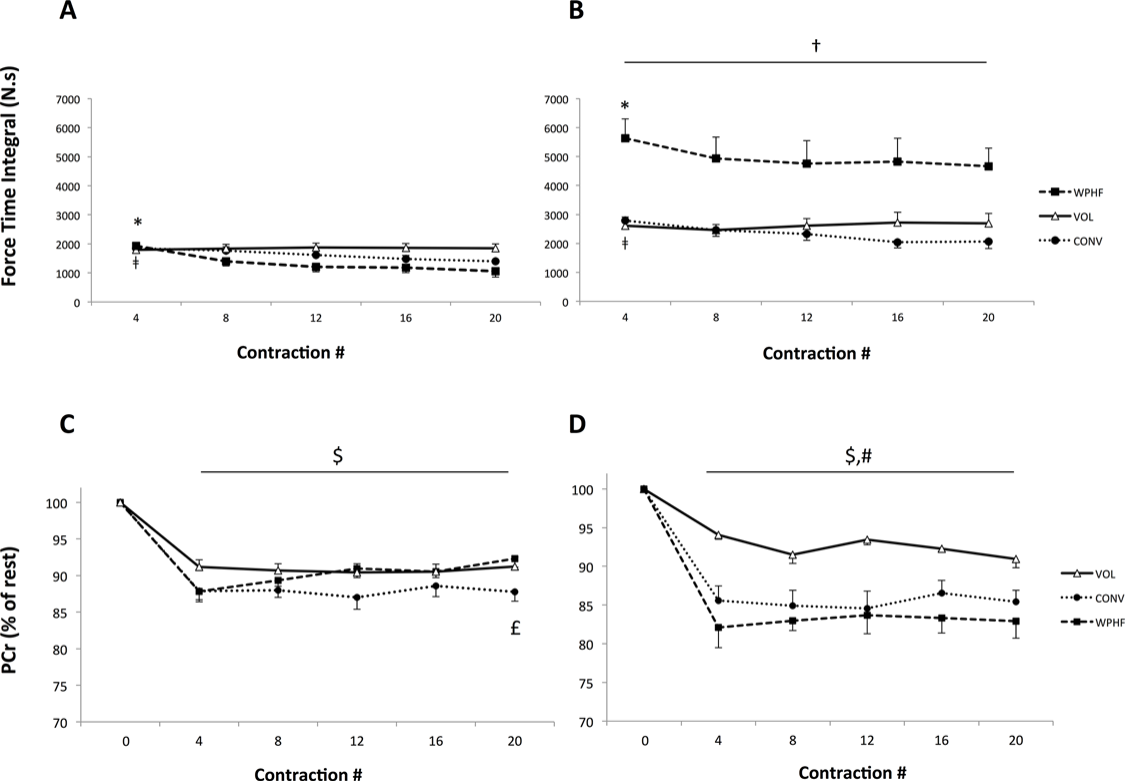
Force time integral and PCr concentrations expressed in percentage of the resting values (i.e., C0) for the non-responders (A & C) and responders (B & D) for each protocol calculated after contraction C4, C8, C12, C16, C20. * WPHF: C4 significantly different from C8, C12, C16, C20 (P< 0.05), ǂ CONV: C4 significantly different from C12, C16, C20 (P< 0.05), † WPHF for all contractions significantly different from all other exercise modalities illustrated (irrespective of group; P< 0.05). ^$^ Significantly different than C0 for VOL, CONV and WPHF NMES for all contractions (P< 0.05), ^#^ Values for VOL for all contractions significantly higher than CONV and WPHF NMES (P< 0.05), ^£^ C20 for CONV significantly lower than C20 for both VOL and WPHF (P < 0.05). Results are presented as means ± SE.

Fig 3 depicts for one non-responder and one responder subjects, the force profiles for the first four and the last four contractions of WPHF, CONV and VOL protocols. With regard to FTI time course, a significant group × protocol interaction was found. For all the contractions (C4—C20), the average FTI of WPHF in the responder group was significantly higher (P < 0.05) as compared to the other exercise modalities within the same group and significantly higher than all exercise modalities within the non-responder group. Moreover, a significant protocol × contractions interaction was observed (Fig 4A and 4B). For both the responder and non-responders, WPHF-induced FTI was significantly higher for the initial contractions (C4) than for the subsequent ones (C8, C12, C16, C20). With regard to the CONV protocol, both groups showed higher FTI values for C4 as compared to C12 (P = 0.06), C16 and C20 (P < 0.05). The VOL protocol showed no significant time-related changes in FTI.

A significant group x protocol interaction was found for the fatigue index. In the non-responder group, WPHF induced a higher fatigue (- 43 ± 24%) as compared to CONV (-25 ± 12%). In contrast, for the responders, the fatigue index did not differ significantly between the two stimulation protocols (WPHF: -16 ± 16% vs. CONV -25 ± 15%). Irrespective of the group, VOL showed no force loss over time resulting in a significantly lower fatigue index when compared to the two NMES conditions. When comparing groups, WPHF-induced fatigue was significantly lower in the responders than in the non-responders whereas CONV led to identical fatigue levels.

The NMES current intensity required to obtain 10% MVC was significantly higher for CONV as compared to WPHF (P < 0.05) with no difference between groups (P > 0.05), neither for WPHF (non-responders: 18 ± 5 mA *vs*. responders: 15 ± 5 mA) nor for CONV (non-responders: 110 ± 19 mA *vs*. responders; 122 ± 19 mA).

### ^31^P-MRS

Both resting PCr and Pi concentrations as well as resting Pi/PCr ratio were not significantly different between protocols and groups (Table 1). When considering the changes of relative PCr concentration (% of rest) over time, both the non-responders and responders showed a significant decline at all contractions (C4, C8, C12, C16, C20) as compared to rest (C0) for all three protocols (Fig 4C and 4D). No significant differences were found in between those contractions, i.e., for all protocols PCr plateaued after C4 until C20. The group x protocol interaction showed that for the responder group, PCr depletion was significantly higher for NMES as compared to VOL for all contractions. The only group x protocol x contraction interaction was found in the non-responders who showed a significantly higher (P< 0.05) PCr depletion for CONV as compared to VOL and WPHF after C20. A significant effect of protocol was found for both delta PCr and end-protocol Pi/PCr with higher values for NMES as compared to VOL (Table 1).

**Table 1:** Resting values and changes from rest (delta) for PCr, Pi/PCr and pH.

		WPHF	CONV	VOL
***Responders***	Resting PCr (mM)	20.7 ± 5.4	22.7 ± 6.2	21.8 ± 5.9
	Resting Pi (mM)	5.2 ± 1.3	5.4 ± 1.3	5.0 ± 1.4
	Resting Pi/PCr	0.34 ± 0.06	0.33 ± 0.05	0.31 ± 0.05
	Resting pH	6.95 ± 0.04	6.95 ± 0.02	6.97 ± 0.03
	Delta PCr (mM)	3.4 ± 0.9	3.2 ± 1.0	1.7 ± 0.7^§^
	End-protocol Pi/PCr	0.53 ± 0.12	0.51 ± 0.05	0.40 ± 0.05^§^
	Delta pH	-0.03 ± 0.04	0.00 ± 0.04	-0.01 ± 0.02
***Non-responders***	Resting PCr (mM)	23.9 ± 7.1	22.5 ± 6.6	24.2 ± 5.8
	Resting Pi (mM)	5.4 ± 1.5	4.9 ± 1.3	5.1 ± 1.3
	Resting Pi/PCr	0.35 ± 0.10	0.34 ± 0.07	0.32 ± 0.08
	Resting pH	6.98 ± 0.04	6.97 ± 0.04	6.98 ± 0.03
	Delta PCr (mM)	2.4 ± 1.2	2.9 ± 1.6	2.3 ± 1.0^§^
	End-protocol Pi/PCr	0.38 ± 0.08	0.44 ± 0.08	0.39 ± 0.07^§^
	Delta pH	0.01 ± 0.04	-0.01 ± 0.03	0.00 ± 0.03

^§^ Significantly different from NMES (P< 0.05), independently of the group.

**Fig 5:**
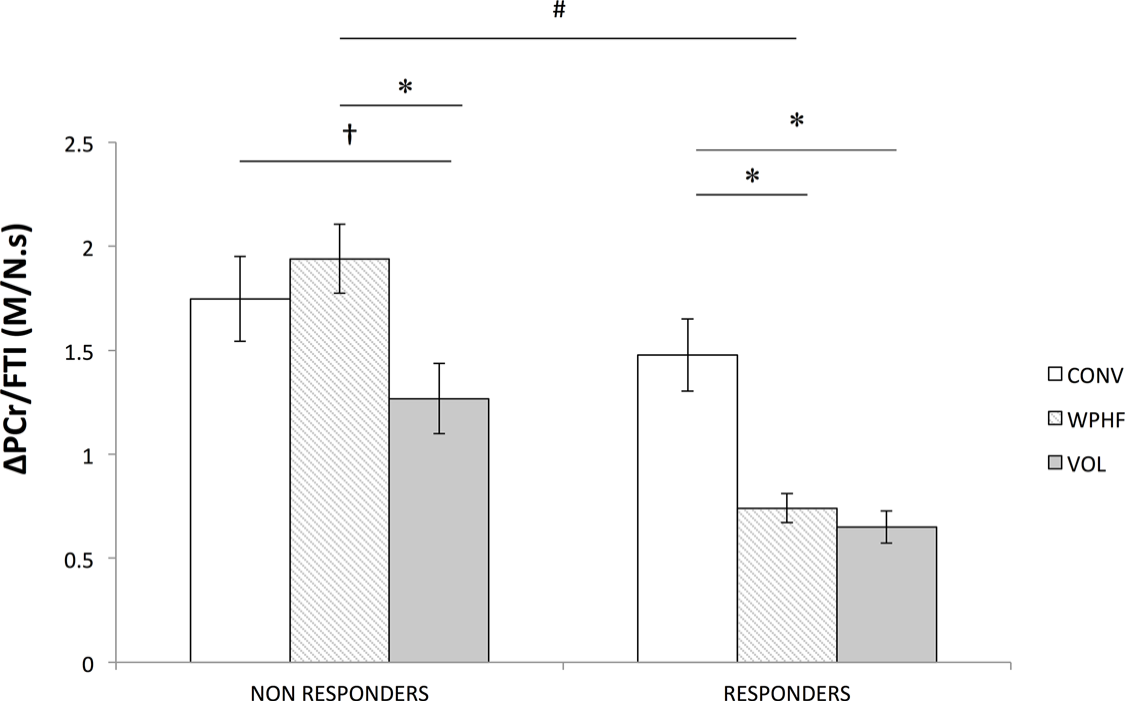
ΔPCr/FTI (in M/N.s) ratio for the non-responders and responders for each protocol. * Significantly different within groups for P < 0.05, † non-significant tendency (P = 0.07), # WPHF significantly different between groups for P < 0.05. Results are presented as means ± SE.

A significant effect of protocol was noted for the ΔPCr/FTI ratio so that lower values were obtained for VOL (P< 0.05) compared to the two NMES protocols. Interestingly, a significant group × protocol (P < 0.05) interaction was observed for the ΔPCr/FTI ratio (Fig 5). For the responders, this ratio was significantly lower for WPHF than for CONV (P < 0.05) with a statistical power of 0.88. On the contrary, in the non-responder group, ΔPCr/FTI ratio was not significantly different (P >0.05) between CONV and WPHF. When comparing the ΔPCr/FTI ratio of WPHF between groups, responders showed a significant lower metabolic demand (P < 0.05) compared to non-responders with a statistical power of 0.87.

Resting intracellular pH values were not significantly different between groups and protocols (Table 1). Also, no main effect or interaction (P > 0.05) was observed for ΔpH (Table 1).

## Discussion

In the present study, we compared the force output and metabolic changes associated to two NMES protocols (CONV and WPHF) and a series of VOL plantar flexions. We demonstrated that for the responder group (i.e., a subset of subjects displaying higher force levels so called “Extra forces”), the decline in FTI during the WPHF protocol was less pronounced as compared to the non-responders. Moreover, for the responders, the corresponding energy demand was comparable to that of VOL contractions and substantially lower than the metabolic demand of CONV.

### Responders

The EF occurred during the WPHF protocol only in 7 out of 18 subjects that were identified as responders. For this subgroup, the total FTI was four times higher for WPHF when compared to CONV whereas the relative mean force (∼20% MVC) was twice as high as compared to CONV (9.6% MVC). These results are in accordance with the isometric force increment reported in previous studies that investigated the *triceps surae* in similar conditions [15, 19, 22, 36]. Interestingly, in terms of metabolic demand, the ΔPCr/FTI ratio was three times lower for both WPHF and VOL as compared to CONV in the responders. Thus, both WPHF and VOL protocols exhibited an identical energy demand, which was remarkably lower than that of CONV. From this it could be inferred that the lower metabolic demand of WPHF could hint to a reduced number of activated fatigable, fast-twitch fibers and a larger proportion of oxidative, slow-twitch fibers activated via afferent pathways as suggested previously [5, 17, 19]. The hypothesis that WPHF recruits motor units in resemblance to the size principle rather than in a random order is based on the fact that longer pulse durations preferentially depolarize sensory axons having a lower activation threshold [13, 14]. Furthermore, the low current intensities of WPHF (approximately 15% of CONV intensity in the present study) could have minimized the antidromic volley thus maximizing sensory input. Consequently, contrary to CONV, a synaptic recruitment of motor units could have been mediated by WPHF via the activation of large afferent fibers. As a result, the activation of afferent fibers at high stimulation frequencies would have increased the number of recruited motoneurons in a size order from small to large due to a temporal summation of Ia excitatory postsynaptic potentials in the motoneurons [20]. A preferential recruitment of small motoneurons in response to WPHF could therefore explain the lower metabolic demand observed in the responders.

The level of mechanical fatigue we observed might further support the assumption that slow-twitch fibers were additionally recruited via afferent pathways during WPHF but not during CONV. Despite the higher overall mechanical output during WPHF and the high frequencies applied, the fatigue index shows a comparable force loss for the NMES protocols (WPHF: -16% vs. CONV: -25%). Our findings suggest that pattern of muscle activation associated with WPHF for the responders might approach the physiological recruitment strategy of VOL contractions according to the size principle, leading to a reduced metabolic demand as compared to CONV protocol.

### Non-responders

The mechanical and metabolic responses to WPHF showed large inter-individual differences. Indeed, the majority of our sample, i.e., 11 out of 18 subjects did not respond to WPHF as illustrated by similar FTI values for the three protocols. Moreover, both NMES protocols led to a higher metabolic demand as compared to VOL. In contrast to the responder group, no WPHF-induced synaptic activation of motor units can be assumed in the non-responders when considering the FTI kinetics and the metabolic demand. Due to the consistent absence of EF, FTI for WPHF did not exceed that of CONV and VOL at any time-point. The metabolic demand (i.e., ΔPCr/FTI ratio) of the evoked contractions was equivalent between the two NMES modalities, showing higher values than VOL contractions. From the findings it could be assumed that during both NMES protocols muscle activation (i.e., the type of activated motor units) could have been similar in such a way that mainly motor axons were recruited. As a consequence, the high frequency applied and the reduced number of motor units recruited (resulting from the 6 times lower stimulation intensity than that needed for CONV) might have caused greater fatigue for WPHF (-43%) as compared to CONV (-25%) in the non-responders. These results are in accordance with those of Neyroud et al. who reported that the FTI for WPHF was 1.7 fold smaller as compared to CONV NMES [27]. The potential mechanism underlying this pronounced fatigue for WPHF could be activity-dependent hyperpolarization, a phenomenon to which motor axons are particularly susceptible [13]. Therefore, especially in the non-responders, activity-dependent hyperpolarization might have increased motor axonal excitability threshold and led to a loss of solicited motor units and reduced mechanical output, respectively. To conclude, in the absence of EF generation, WPHF resulted in greater fatigue as compared to CONV and was unable to reduce the metabolic demand of the contraction. This let us to assume that motor unit recruitment was similar for both NMES protocols in the non-responders and was less effective than VOL.

### Responders vs. non-responders

The low occurrence of responders (~40%) is in contrast with previous studies reporting a responder percentage of 85–100% [15, 17, 36] and in agreement with recent findings [21, 22, 27]. The underlying causes of the high inter-individual variability in force output during WPHF have not yet been sufficiently investigated. As previously mentioned, we hypothesize differences in muscle activation pattern between groups. In the non-responders, motor axons might have been directly recruited, thereby resulting in antidromic collision and a more pronounced mechanical fatigue. This observation is in line with previous studies showing higher muscle fatigue when motor axons were activated at high frequencies [25, 37]. Based on the same calculation of the fatigue index, Gorgey et al. [37] reported a significantly higher muscle fatigue for stimulation at 100 Hz compared to 25 Hz (76% vs. 39%, respectively) at an intensity corresponding to 75% MVC. In addition, Matheson et al. [25] showed that the acidosis and the Pi/PCr ratio increased with stimulation intensity (and therefore with the number of activated motor axons) but levelled off for stimulation frequencies higher than 30 Hz, likely due to impaired membrane excitability. Overall, the preferential activation of efferent pathways in the non-responders exacerbates the magnitude of muscle fatigue.

Indeed, the activation of afferent pathways in responders might have led to a synaptic recruitment of motor units and less pronounced fatigue. Since sensory axonal activation depends on the distance from the stimulating electrodes to the axons as well as on axonal diameter [38], one could assume a preferential activation of sensory fibers in the responder group due to particular axon characteristics (such as size) and/or a favorable orientation or dispersion of these nerve axons relative to NMES electrodes. Moreover, neuromodulator activity within neural circuits might have further enhanced the high variability of EF between subjects in response to WPHF [39].

### CONV vs. VOL

Consistent with former ^31^P-MRS studies, we showed a systematically higher metabolic demand for CONV than for VOL [12, 28, 29]. In terms of exercise characteristics (relative intensity, duration and duty cycle) our protocol design most closely resembles that of Vanderthommen et al. [28]. In our current study, PCr depletion for CONV was less pronounced (12–15%) as compared to this previous investigation (40%). The more severe PCr consumption observed by Vanderthommen et al. [28] could be explained by the fact that they investigated the quadriceps femoris—which is a muscle containing approximately the same proportion of slow and fast fibers—while we considered the predominantly slow *triceps surae* in the present study [40]. Moreover, in the former study stimulation frequency was twice as high as in our study (50 *vs*. 25 Hz) and NMES current intensity was progressively increased to maintain a constant force output, which may have resulted in a higher metabolic demand [24, 25]. Also, due to the very short pulse duration of 50 μs applied in the present study, phase charge was up to 6 fold lower in our experiments (5.75 x 10^−6^ C) as compared to that induced by previous CONV protocols (13.5–38 x 10^−6^ C) which used longer pulse duration (i.e., 200–400 ms) [12, 28].^.^ These methodological differences between different studies might also explain the fact that, contrary to Vanderthommen et al. [28] (ΔpH of 0.3–0.4) and Jubeau et al. [12] (ΔpH of 0.2–0.3) our NMES protocols did not result in metabolic acidosis so that the energy supply was preferentially provided by oxidative mechanisms [41]. Overall, our results confirm the previously reported metabolic differences between CONV and VOL which are mainly due to the non-physiological motor unit recruitment of CONV, being temporally synchronous [8, 9], spatially restricted [9, 29] and non-selective [7–10].

### Limitations

Considering the different contribution of sensory and motor axons to force production between the two NMES modalities [22] and the different magnitude of sensory inputs between the *soleus* and *gastrocnemii* muscles [42], the spatial distribution of motor unit recruitment may vary between WPHF and CONV. For instance, one could hypothesize that the *soleus* might have been relatively more active than the *gastrocnemii* during WPHF. Although the size of our ^31^P surface coil (120 x 140 mm loop and a 240 x 120 mm butterfly coil) allows to get a representative sample of the overall muscle composition—comprising both superficial and deep *triceps surae* muscle portions [43]—the non-localized acquisition scheme we used did not permit to discriminate between the metabolic contribution to EF of the *gastrocnemii* and the *soleus* muscles. As a result, the contribution of the soleus to the overall metabolic variations might have been underestimated as compared to the superficially-located *gastrocnemii* muscles. Localized spectroscopic techniques [12, 43] would be of interest to compare the metabolic activity among the plantar flexor muscles in responders.

### Practical Use of WPHF

Contrary to previous studies that tended to discourage the use of high-pulse frequencies for rehabilitation purposes–in an attempt to prevent muscle fatigue [37, 44],–force increases induced by WPHF could prove beneficial to enhance muscle mass and function without causing a higher fatigue than CONV. However, the low responder percentage and the high EF variability between and within individuals should be considered as a constraint when integrating WPHF in practical use.

Further studies are needed to fully understand the mechanisms underlying the EF generation and to compare the type of recruited motor units between individuals. Stimulation over the nerve trunk could prove useful to investigate and quantify the contribution of afferent pathways for both groups at a given submaximal stimulation intensity. One could hypothesize that for a given contraction intensity (i.e., 5–10% MVC) the type of recruited motor units would differ between responders and non-responders (which would become manifest in different H-reflex and M-wave amplitudes between groups). Potentially, non-responders could be turned into responders by lowering the stimulation intensity in order to reduce antidromic collision in these subjects. In this context, Dean et al. [45] have recently shown that even at stimulation intensities below sensory and motor threshold, synaptic input could be transformed into a motor response in 7 out of 9 subjects (i.e. 78% of all subjects). Assuming that the responder percentage could be increased, WPHF would be in particular advantageous for patients that are highly vulnerable to fatigue. However, patients with neural disorder might show a different response in EF occurrence, magnitude and variability. Recently however, the EF phenomenon has been reported in patients suffering from stroke [45] and cerebral palsy [21].

## Conclusions

We investigated muscle energetics for the relatively unexplored stimulation modality WPHF. Only for the responder group (constituting 40% of the sample tested), modifying the stimulation parameters from CONV into WPHF resulted in a lower metabolic demand, which was comparable to VOL contractions. Moreover, fatigue was less pronounced for WPHF in the responders as compared to the non-responders. All these observations point to an additional recruitment of slow-twitch, fatigue-resistant motor units via afferent pathways with WPHF, likely in accordance with the size principle.
